# ASO Author Reflections: Tumor Size as a Determinant of Anatomic Resection Benefit in Intrahepatic Cholangiocarcinoma

**DOI:** 10.1245/s10434-025-17315-8

**Published:** 2025-04-13

**Authors:** Jun Kawashima, Miho Akabane, Timothy M. Pawlik

**Affiliations:** 1 Department of Surgery, The Ohio State University Wexner Medical Center and James Comprehensive Cancer Center, Columbus, OH USA; 2 Department of Gastroenterological Surgery, Yokohama City University School of Medicine, Yokohama, Japan; 3 Department of Surgery, The Urban Meyer III and Shelley Meyer Chair for Cancer Research, Columbus, OH USA; 4 Oncology, Health Services Management and Policy, Wexner Medical Center, The Ohio State University, Columbus, OH USA

## Past

While achieving an R0 resection margin remains the primary goal of hepatic resection, the optimal extent of resection for intrahepatic cholangiocarcinoma (ICC) remains unclear.^1^ Specifically, the comparative oncologic benefits of anatomic resection (AR) versus non-anatomic resection (NAR) for patients with ICC have not been well established, as there are conflicting data from small retrospective studies.^1,2^ In the setting of hepatocellular carcinoma, a survival advantage of AR over NAR has been demonstrated; however, the benefit may be specific to patient subgroups depending on tumor size, histological differentiation, and microvascular invasion influencing the therapeutic benefit of AR.^3^ In the current study, we hypothesized that tumor morphology may modulate the survival benefit of AR versus NAR among patients undergoing resection of ICC.

## Present

Tumor size is a well-established prognostic factor for ICC, reflecting tumor biology and serving as one of the few clinicopathologic variables that can be reliably assessed preoperatively.^4^ The current study evaluated the impact of tumor size on the relative oncologic benefit of AR versus NAR among patients with ICC undergoing hepatic resection. We analyzed 969 patients who underwent upfront curative-intent resection with R0 margin for solitary ICC at multiple international institutions. Among these individuals, 506 (72.9%) underwent AR, while 263 (27.1%) had a NAR. On multivariable analysis for recurrence-free survival (RFS), there was an interaction between tumor size and AR [hazard ratio (HR) 0.94, 95% confidence interval (CI) 0.88–1.00, *p* = 0.045], suggesting that tumor size was a key determinant in selecting AR over NAR for ICC. A plot of the interaction demonstrated that AR was associated with improved RFS for tumors ≥ 4.0 cm. Of note, among 257 (26.5%) patients with tumors < 4.0 cm, RFS was no different relative to NAR versus AR (3-years RFS: 65.2%, 95% CI 55.7–76.2 vs. 58.1%, 95% CI 49.2–68.5; *p* = 0.720). In contrast, among 712 (73.4%) patients with tumors ≥ 4.0 cm, AR was associated with improved RFS (3-years RFS: 34.7%, 95% CI 27.5–43.8 vs. 44.9%, 95% CI 40.4–50.0; *p* = 0.018). In addition, the 4.0 cm tumor size threshold was applicable to both patients with early (stage I) and advanced stage (stage II/III) features that were identified by pathological examination. These findings suggest that tumor size may be a valuable preoperative criterion in surgical decision-making relative to AR versus NAR for patients with ICC.

## Future

As treatment strategies for ICC continue to evolve, the role of repeat liver resection following recurrence has gained increasing attention as a promising therapeutic option.^5^ Given that repeat liver resection may offer survival benefits for selected patients, the initial surgical approach should be planned to optimize oncologic outcomes. Data from the current study suggested that AR did not provide a survival advantage over NAR for tumors < 4.0 cm in terms of RFS. As such, preserving liver parenchyma with NAR among patients with smaller ICC may be a reasonable approach. In contrast, AR may be more important for larger tumors measuring ≥ 4.0 cm as AR was associated with better RFS in this setting. Overall, the data highlight the need for an individualized decision-making approach to patients with ICC. Moving forward, surgical strategies should integrate tumor morphology and biology to refine selection criteria for AR and NAR among patients with ICC.
